# Transcription Factor HBP1 Enhances Radiosensitivity by Inducing Apoptosis in Prostate Cancer Cell Lines

**DOI:** 10.1155/2016/7015659

**Published:** 2016-01-28

**Authors:** Yicheng Chen, Yueping Wang, Yanlan Yu, Liwei Xu, Youyun Zhang, Shicheng Yu, Gonghui Li, Zhigeng Zhang

**Affiliations:** ^1^Department of Urology, Sir Run-Run Shaw Hospital, College of Medicine, Zhejiang University, Hangzhou 310016, China; ^2^Department of Urology, Wuyi First People's Hospital, Zhejiang 321200, China

## Abstract

Radiotherapy for prostate cancer has been gradually carried out in recent years; however, acquired radioresistance often occurred in some patients after radiotherapy. HBP1 (HMG-box transcription factor 1) is a transcriptional inhibitor which could inhibit the expression of dozens of oncogenes. In our previous study, we showed that the expression level of HBP1 was closely related to prostate cancer metastasis and prognosis, but the relationship between HBP1 and radioresistance for prostate cancer is largely unknown. In this study, the clinical data of patients with prostate cancer was compared, and the positive correlation was revealed between prostate cancer brachytherapy efficacy and the expression level of HBP1 gene. Through research on prostate cancer cells in vitro, we found that HBP1 expression levels were negatively correlated with oncogene expression levels. Furthermore, HBP1 overexpression could sensitize prostate cancer cells to radiation and increase apoptosis in prostate cancer cells. In addition, animal model was employed to analyze the relationship between HBP1 gene and prostate cancer radiosensitivity in vivo; the result showed that knockdown of HBP1 gene could decrease the sensitivity to radiation of xenograft. These studies identified a specific molecular mechanism underlying prostate cancer radiosensitivity, which suggested HBP1 as a novel target in prostate cancer radiotherapy.

## 1. Introduction

Prostate cancer is very common in western countries, the incidence of which ranks first in male cancers [1]. With increased life expectancy and the change of diet and lifestyle, the incidence and mortality rate of prostate cancer are rising in China, which is particularly prominent in some economically developed regions [2, 3]. As such, studies on prostate cancer pathogenesis and therapy are prevalent.

In the past, radical prostatectomy was preferred clinically for localized prostate cancer of early stage and low-risk patients [4], while for late stage, intermediate- and high-risk patients, androgen deprivation therapy has been used [5]. In the last 20 years in western countries, radiotherapy (including EBRT and permanent radioactive seed implantation) for the treatment of prostate cancer has gradually replaced the radical prostatectomy and androgen deprivation therapy and has become one of the main methods for the treatment of prostate cancer [6, 7]. Radiotherapy for prostate cancer has been gradually carried out in China in recent years with good effect. However, biochemical recurrence or clinical relapse occurred in some patients after radiotherapy, potentially due to acquired radioresistance [8, 9]. While the mechanism of radioresistance for prostate cancer is unclear, the methodology into the research and treatment for prostate cancer radioresistance is lacking. Studies that focus on the reduction of prostate cancer cell resistance and promote increased tumor cell sensitivity to radiotherapy treatment are predominant in prostate cancer.

HBP1 (HMG-box transcription factor 1) is a transcriptional inhibitor belonging to HMG-box transcription factor family and could inhibit the expression of a number of growth regulatory genes and oncogenes [10]. Our recently published study showed that the expression level of HBP1 in prostate cancer cells and prostate cancer tissues was significantly lower than the normal cells and normal adjacent matched tissues, respectively. Lower expression level of HBP1 resulted in the increased expression level of MIF gene (macrophage migration inhibitory factor) and promoted the ability of colony formation and invasion of prostate cancer cells. Though the expression level of HBP1 in prostate cancer is closely related to metastasis and prognosis [3], the relationship between HBP1 and prostate cancer radiotherapy is unknown.

Therefore, we hypothesized that decreased expression of HBP1 in prostate cancer leads to radioresistance. In this study, we combined clinical data of patients with prostate cancer, prostate cancer cells, and animal models to analyze the relationship between HBP1 gene and prostate cancer radiosensitivity. Furthermore, we determined the relationship between HBP1 and apoptosis during prostate cancer radiotherapy. These studies clarify the role of HBP1 in prostate cancer radiotherapy and identify HBP1 as a new biomarker in prostate cancer radiotherapy as well as a novel target for radiosensitization.

## 2. Materials and Methods

### 2.1. Patient Information

Sample collection was in accordance with the terms of the Medical Ethical Committee of the Zhejiang University and followed the guidelines of the Declaration of Helsinki. Blood samples from 66 patients with low-risk and intermediate-risk prostate cancer treated with brachytherapy were obtained from the Department of Urology, Sir Run-Run Shaw Hospital (Zhejiang University, China) from February 2009 to February 2011. The inclusion criteria are National Comprehensive Cancer Network (NCCN) risk grouping criteria, which are low-risk patients, defined as Gleason score < 6, prostate-specific antigen (PSA) < 10 ng/mL, and T1c-T2a, and intermediate-risk patients, defined as Gleason score = 7, PSA 10–20 ng/mL, and T2b-T2c. The age of patients was ranged from 69 to 87; the median age was 78 years. All patients were diagnosed by prostate needle biopsy, according to the Gleason score system: the lowest score is 3 + 3 = 6, and the highest is 4 + 3 = 7; the lowest serum PSA before treatment was 2.86 ng/dL, and the highest was 19.3 ng/dL. All patients underwent prostate magnetic resonance imaging and the whole body bone PET/CT examination, and no bone metastases were found in any patient. All patients underwent the implantation of ^125^iodine (^125^I) particles according to the guidelines of American Society for Radiation Oncology (ASTRO) and American College of Radiology (ACR). The three-dimensional image is reconstructed of the prostate; Brachy probpsstep V3.02 planning software was applied to develop treatment plans and the expected target dose is 144 Gy. The median number of particles was 64, with a minimum of 54 and a maximum of 91. The implantation condition was checked via pelvic CT scan on the first day after operation, calculating the value of D90. In 66 patients, the median D90 value was 142 Gy, with a minimum of 122 Gy and a maximum of 151 Gy. Patients were discharged after three days and the catheter was removed after one week. The blood samples were collected from patients before and after radiation therapy. Full ethical consent was obtained from all patients.

### 2.2. Cell Culture

DU-145 cell lines (prostate carcinoma cell lines) were purchased from the American Type Culture Collection (ATCC; Manassas, VA, USA). All cells were cultured in Dulbecco's modified Eagle's medium (DMEM; Gibco, Grand Island, NY) containing 10% fetal bovine serum (Hyclone, Logan, UT), supplemented with 50 units of penicillin/streptomycin.

Irradiation was carried out using 6 MV X-rays generated by a linear accelerator (PRIMUS-M, Siemens) at a dose rate of 2 Gy/min. During irradiation, cells were put at room temperature with 2 cm thick tissue glue placed on the culture dish and irradiated by X-rays at a source distance of 100 mm.

### 2.3. Plasmid Construction

The pBaBE-HBP1 expression vectors were a kind gift from Dr. Amy S. Yee [11]; pBabe Vector was used as the control. Retroviral gene transduction was carried out using Phoenix packaging cells. RNAi-mediated HBP1 knockdown was accomplished by shRNA produced by the DNA-based shRNA-expressing retroviral vector (pSuper-Retro). The vectors were a kind gift from Dr. Amy S. Yee. The knockdown experiment was performed as described previously [3]. The HBP1 shRNA target sequence is ACTGTGAGTGCCACTTCTC as the pGenesil-HBP1-shRNA plasmid and pGenesil-scramble plasmid were used as the control.

### 2.4. Real-Time PCR

Total RNA was isolated from tissues or cells using TRIzol reagent (Invitrogen, Carlsbad, USA) according to the manufacturer's instructions. Reverse transcription was performed with an iScript cDNA synthesis kit (Bio-Rad) in 20 *μ*L reaction volume containing 1 *μ*g of total RNA. Real-time PCR was performed with Power SYBR Green PCR Master Mix (Applied Biosystems) using a 7500 real-time PCR System (Applied Biosystems). The primers for cyclin D (CCND) (sense: 5′-TGGAGCCCGTGAAAAAGAGC-3′; anti-sense: 5′-TCTCCTTCATCTTAGAGGCCAC-3′), MIF (sense: 5′-GCGGGTCTCCTGGTCCTTCTG-3′; anti-sense: 5′-GTGGGTCCCTGCGGCTCTTA-3′), N-Myc (sense: 5′-ACTTCTACTTCGGCGG-3′; anti-sense: 5′-TCTCCGTAGCCCAAT-3′), and GAPDH (sense: 5′-CACCAGGGCTGCTTTTAACTC-3′; anti-sense: 5′-GAAGATGGTGATGGGATTTC-3′) were designed with Primer Premier Version 5.0 software and their efficiency was confirmed by sequencing their conventional PCR products.

### 2.5. Western Blots

Proteins extracted from cells were quantified by the modified Lowry method. Discontinuous sodium dodecyl sulphate-polyacrylamide gel electrophoresis (SDS-PAGE) was performed using Bio-Rad mini-protein II electrophoresis and transferred into polyvinylidenedifluoride membrane (PVDF, Amersham Pharmacia Biotech, Piscataway, NJ). Protein extract was electrophoresed and electroblotted onto supported nitrocellulose membranes. Blots were blocked for 2 h with 5% nonfat dry milk in Tris-buffered saline (TBS) at room temperature for 1 h and then incubated with anti-HBP1 and anti-GAPDH (cat. sc-8488 and sc-32233, Santa Cruz Biotechnology, Santa Cruz, CA, USA) specific primary antibodies (1 : 1000 dilution) at room temperature for 2 h. Blots were incubated with HRP-conjugated secondary anti-rabbit antiserum (Santa Cruz, CA, USA, 1 : 5000 dilution in TBS) for 1 h. After several washes with 0.1% TBS-Tween 20, immunoreactive proteins were visualized with enhanced chemiluminescence (ECL) and captured on an X-ray film. Protein levels were quantified using Biosense 300 software (Oberhaching, Germany). Relative protein expression level was calculated by comparing with the expression of controls.

### 2.6. MTT Analysis

Chondrocytes were seeded in a 96-well culture plate. After 12 h, cells were treated with diosgenin (0, 10, 50, and 100 *μ*M) for 24 h. The cells were incubated with MTT solution (5 mg/mL) at 37°C for 4 h and then with dimethylsulfoxide (DMSO) by shaking at room temperature for 10 min. The spectrophotometric absorbance was measured at 570 nm on a multifunctional microplate reader (Tecan, Durham, NC, USA).

### 2.7. Flow Cytometry Analysis for Apoptosis

The percentage of apoptotic cells was assayed by the Annexin-V-FLUOS Staining Kit (Roche). Briefly, 1 × 10^5^ cells were seeded in six-well plates and cultured for 24 hours. The cells were collected and resuspended in 100 *μ*L binding buffer. Then, the cells were incubated with 5 *μ*L FITC-Annexin-V in the dark for 15 minutes at room temperature. Subsequently, 5 *μ*L PI was added and incubated with the cells for 20 minutes at room temperature in the dark. Finally, the cell samples were examined in the flow cytometer. Each assessment of proliferation and apoptosis was repeated three times.

### 2.8. Xenograft Model

All animal care and experimental protocols in this study were approved by the animal ethics committee of Zhejiang University, China. Male BALB/c nude mice (4~6 weeks old) were purchased from Weitong Lihua Experimental Animal Technical Company (Beijing, China). Approximately 5 × 10^7^ cells in 0.1 mL of PBS were subcutaneously injected in the right thigh of nude mice and treatment was started when the tumors reached an average volume of 50 mm^3^. Mice bearing similar tumor volumes were chosen and randomly divided into 2 groups for irradiation with 3 mice in each group: Con shRNA group and HBP1 shRNA group. Mice were checked daily for mortality relevant to treatment. Body weight and tumor size were measured every 3 days. Mice were sacrificed three weeks after treatment and tumor volumes were calculated using the formula: tumor volume (mm^3^) = [length (mm) × width (mm)^2^]/2.

### 2.9. Statistical Analysis

All experiments were repeated three times with independent cultures and similar results were obtained. Data are presented as the mean and 95% confidence interval (CI). Statistical significance was determined by the two-tailed unpaired Student's *t*-test. *P* values less than 0.05 were considered significant.

## 3. Results

### 3.1. Positive Correlation between Prostate Cancer Brachytherapy Efficacy and the Expression Level of HBP1 Gene

**Figure 1:**
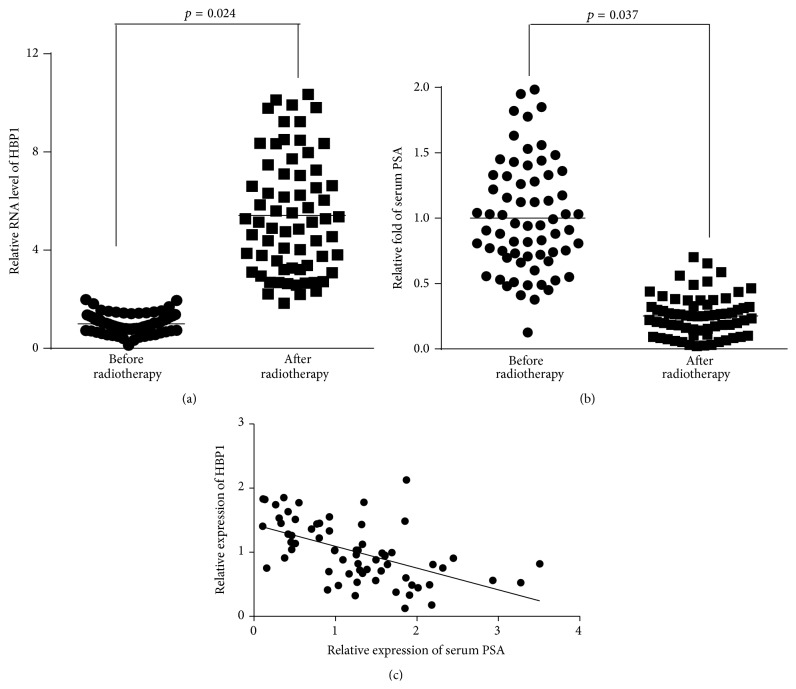
The relationship between prostate cancer brachytherapy efficacy and the expression level of HBP1 gene. (a) RT-PCR assay was used to detect the expression level of HBP1 gene in 66 pairs of blood samples of prostate cancer patients before or after brachytherapy. (b) The serum PSA level from patients was detected in 66 pairs of blood samples of prostate cancer patients before or after brachytherapy. (c) The relationship between the expression level of HBP1 and PSA was analyzed in 66 prostate patients who received brachytherapy.

The expression level of HBP1 gene in blood samples was detected via RT-PCR in 66 patients who received radiotherapy and compared to preradiation control samples. As shown in Figure 1(a), we found that HBP1 expression level increased in the blood samples of brachytherapy-treated patients compared with pretreatment blood samples. For these patients, PSA levels were as the main criteria to judge the efficacy of brachytherapy; we found that PSA was significantly downregulated in irradiated patient samples (Figure 1(b)). Furthermore, the relationship between the expression level of HBP1 and serum PSA was analyzed, and the statistical results suggested that the expression level of HBP1 was positively correlated with serum PSA; the prognosis of patients with low expression level of HBP1 is poor (Figure 1(c)).

### 3.2. HBP1 Expression Levels Are Negatively Correlated with Oncogene Expression Levels

**Figure 2:**
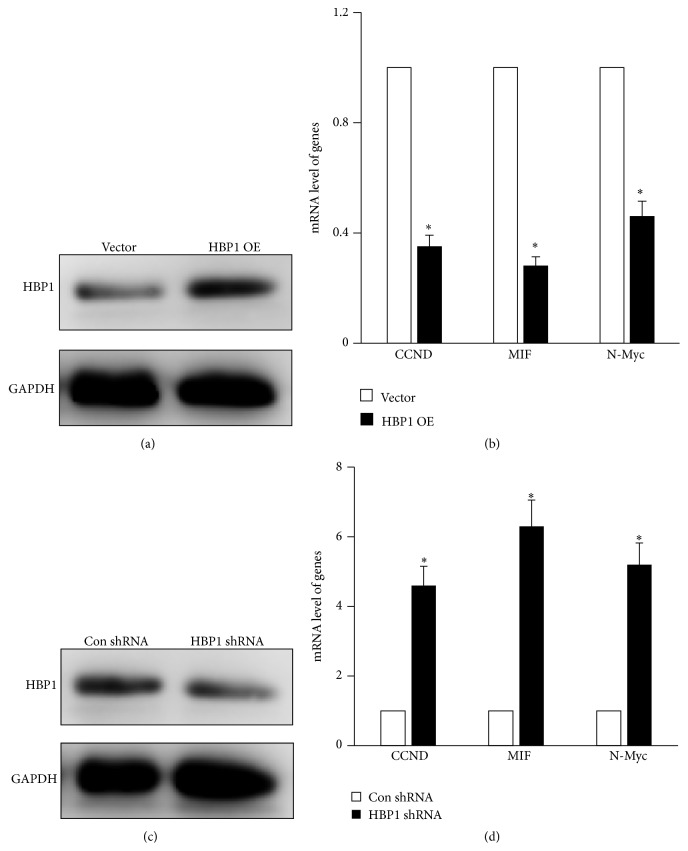
The establishment of prostate cancer cell lines with different expression levels of HBP1 gene. (a) Western blot assay was used to detect the protein level of HBP1 in monoclonal cell lines stably transfected by pBaBE-vector (Vector) and pBaBE-HBP1 (HBP1 OE) plasmid in DU-145 cells. (b) The mRNA level of the HBP1 downstream genes was detected via RT-qPCR including CCND, MIF, and N-Myc in Vector and HBP1 OE group. ^*∗*^ *p* < 0.05 versus control. (c) Western blot assay was used to detect the protein level of HBP1 in monoclonal cell lines stably transfected by pGenesil-scramble (Con shRNA) and pGenesil-HBP1-shRNA (HBP1 shRNA) plasmid in DU-145 cells. (d) The mRNA level of the HBP1 downstream genes was detected via RT-qPCR including CCND, MIF, and N-Myc in Con shRNA and HBP1 shRNA groups. ^*∗*^ *p* < 0.05 versus control.

To examine the function of HBP1 in prostate cancer in response to brachytherapy, monoclonal cell lines stably transfected by pBaBE-vector and pBaBE-HBP1 plasmid were selected with puromycin in DU-145 cells. Western blot assay was employed to detect the expression level, and the result showed that HBP1 was significantly upregulated in pBaBE-HBP1 group (HBP1 OE) compared with pBaBE-vector (Vector) group (Figure 2(a)). Furthermore, the mRNA levels of the HBP1 downstream effector genes were detected; we found that HBP1 overexpression could upregulate the expression of these genes, such as CCND, MIF, and N-Myc (Figure 2(b)). Similarly, monoclonal cell lines stably transfected with pGenesil-scramble and pGenesil-HBP1-shRNA plasmid were selected with G418 in DU-145 cells; the expression level of HBP1 was also verified by western blot, and the result showed that pGenesil-HBP1-shRNA (HBP1 shRNA) could markedly reduce the endogenous expression of HBP1 compared with cells transfected with pGenesil-scramble (Con shRNA) (Figure 2(c)). In addition, the knockdown of HBP1 results in an increase in the mRNA levels of CCND, MIF, and N-Myc (Figure 2(d)).

### 3.3. HBP1 Overexpression Sensitizes DU-145 Cells to Radiation

The four established clonal groups (HBP1 OE, Vector, HBP1 shRNA, and Con shRNA) were irradiated using a dose gradient (from 2 to 6 Gy). At 24 h and 48 h after irradiation, MTT assay was used to assess cell growth. Upon optimization of the experimental conditions, we irradiated cells with 4 Gy for subsequent experiments. The survival rates of all four different groups progressively declined following irradiation; the HBP1 OE cells which experienced an approximate 30% reduction in viability compared with the other three groups (Table 1) were especially affected. However, there was no significant difference observed between the Vector group and HBP1 shRNA group (Table 2). The results suggest that upregulation of HBP1 expression could enhance the short-term apoptotic effects of radiation or reduce radiation-resistant prostate cancer.

### 3.4. Overexpression of HBP1 Increases Apoptosis in DU-145 Cells

**Figure 3:**
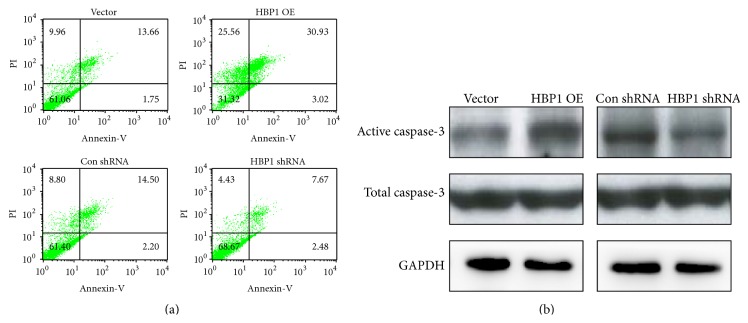
Apoptosis of DU-145 cells with different expression levels of HBP1 gene. (a) Flow cytometry assay was used to detect the apoptosis in Vector, HBP1 OE, Con shRNA, and HBP1 shRNA groups 48 h after irradiation with 4 Gy. (b) Western blot assay was used to detect the activity of proapoptotic protein caspase-3.

The amount of apoptotic cells of the four monoclonal cell lines was measured 48 h after irradiation with 4 Gy by flow cytometry. HBP1 overexpression increased the apoptosis rate compared to the other three groups (Figure 3(a)). Additionally, the activity of proapoptotic protein caspase-3 was detected by western blot in the four different irradiated cell groups. We observed increased caspase-3 levels in HBP1 OE cells, which was consistent with the flow cytometry results (Figure 3(b)).

### 3.5. Sensitivity to Radiation of Xenograft with Different Expression Levels of HBP1 Gene

**Figure 4:**
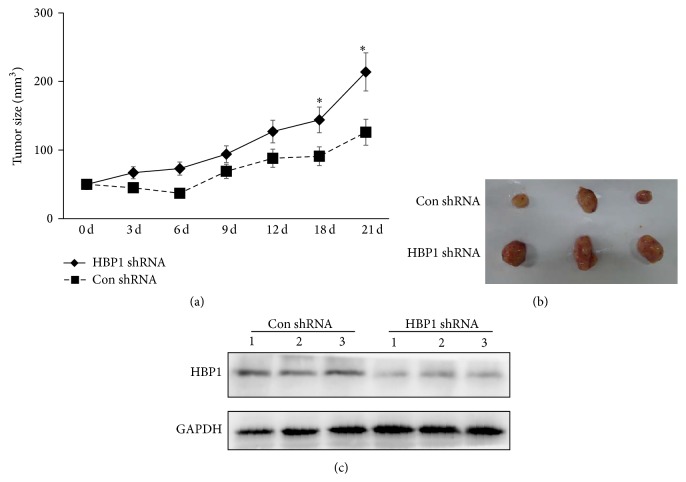
Sensitivity to radiation of xenograft with different expression levels of HBP1 gene. (a) The tumor size was measured in 6-week-old Balb/c male mice in Con shRNA and HBP1 shRNA groups after irradiation. ^*∗*^ *p* < 0.05 versus control. (b) The tumor was removed and observed in Con shRNA and HBP1 shRNA groups after irradiation. (c) The expression of HBP1 was detected via western blot in these tumors from Con shRNA and HBP1 shRNA groups after irradiation.

DU-145 cells from the four stable cell lines were digested to form cell suspensions, which were subcutaneously injected into the right thigh of 6-week-old Balb/c male mice. Tumor formation was observed and measured. Among the nude mice stably inoculated with HBP1 OE cells, viability was severely impaired and these mice were euthanized. Mice bearing tumors derived from the empty vector cells survived, while the specific reasons were not clear (data not shown). When the diameter of tumor reached 0.5 cm, we conducted irradiation sensitivity tests. Among the nude mice stably transfected with Con shRNA cells and HBP1 shRNA cells, the knockdown of endogenous HBP1 could effectively promote the tumor size compared with the Con shRNA cells (Figure 4(a)). When the mice were sacrificed, the tumor was removed and measured; the tumor in HBP1 shRNA group was significantly bigger than Con shRNA group (Figure 4(b)). Furthermore, the expression of HBP1 in tumor was detected via western blot; we found that HBP1 expression was greatly decreased in HBP1 shRNA group (Figure 4(c)). All data suggested that HBP1 had an important role in the development of tumors.

## 4. Discussion

Our preliminary results showed that DU-145 prostate cancer cell lines with high expression level of HBP1 were more sensitive to radiation. According to these results, it can be envisaged that increased expression of HBP1 can enhance the radiosensitivity of prostate cancer cells, whereas reduced expression level of HBP1 in prostate cancer cells is likely to be an important cause of prostate cancer radioresistance. However, additional research is required to support the hypothesis. Furthermore, the mechanism of HBP1 activity is needed to understand its role.

Many studies in cell and animal models have confirmed that HBP1 plays an important role in cell cycle arrest and apoptosis in the regulation of the cell cycle [12, 13]. Apoptosis, as a unique cell death process, plays an important role in irradiation-induced cell death [14]. Apoptosis and overall survival and local recurrence rates are significantly correlated. In patients with advanced cancer, apoptosis of tumor cells increased [15], which was accompanied with increased aneuploid cells [16, 17] and p53 overexpression [18], which could improve the response to radiotherapy of the tumor cells. The local control rate of patients with a high apoptotic index was improved, and distant metastasis was reduced; thus, the prognosis was better. Antiapoptotic mechanism of tumor cells and tumor cell radioresistance are closely linked [14]. Thus, HBP1, which regulates cell cycle progression, likely modulates radiation-induced apoptosis in prostate cancer cells, therefore affecting the efficiency of radiotherapy in prostate cancer cells. At present, there are two main caspase-dependent apoptotic pathways: one is activated by extracellular signals, which triggers intracellular cell death signaling by extracellular death ligand binding to the corresponding death receptor on the membrane and recruiting and activating caspase-2, caspase-8, and caspase-10; the second pathway is activated by intracellular stress signals, primarily mediated by the mitochondria cytochrome C activation that leads to caspase-9 activation [19, 20]. However, the regulation of apoptosis includes a complex series of cascades, involving p53 [21], RB [22], E2F [23], and other factors. The mechanisms of apoptosis vary as greatly as the different tissues themselves. Further study is required to determine if HBP1 is associated with apoptosis and if the interaction is mediated by irradiation.

Presently, while much is known about the upstream regulation of HBP1, little is known about its downstream targets and their roles in radiotherapy in the treatment of prostate cancer. HBP1, as an important transcriptional repressor, may regulate many target genes and signaling pathways. Future experiments to determine the role of HBP1 in apoptosis are worthwhile. New technologies, such as gene chips, could be employed to determine the downstream effectors of HBP1 in prostate cancer radiotherapy.

## 5. Conclusion

Taken together, our data demonstrated that suppression of HBP1 expression not only sensitized prostate cancer cells to radiation but also increased apoptosis in prostate cancer cells. Furthermore, xenograft model was carried on and the result showed that suppression of HBP1 attenuated the sensitivity to radiation in vivo. In addition, the results implied that HBP1 might exert its function via regulating the expression of CCND, MIF, and N-Myc. All these findings have essential implications for the treatment of prostate cancer by radiotherapy and further prospective investigations in a larger patient population might be needed.

## Figures and Tables

**Table 1:** Comparison of cell viability of Vector group and HBP1 OE group at 48 h after different dose gradient radiation.

Dose	Vector group	HBP1 OE group	*p* value
2 Gy	0.590 ± 0.019	0.640 ± 0.014	0.003
4 Gy	0.614 ± 0.015	0.322 ± 0.014	0.001
6 Gy	0.565 ± 0.015	0.292 ± 0.015	0.0002

**Table 2:** Comparison of cell viability of Con shRNA group and HBP1 shRNA group at 48 h after different dose gradient radiation.

Dose	Con shRNA group	HBP1 shRNA group	*p* value
2 Gy	0.530 ± 0.019	0.588 ± 0.023	0.1
4 Gy	0.608 ± 0.006	0.628 ± 0.017	0.08
6 Gy	0.618 ± 0.018	0.595 ± 0.007	0.1
